# Cognitive Impairment in Cerebral Small Vessel Disease Is Associated with Corpus Callosum Microstructure Changes Based on Diffusion MRI

**DOI:** 10.3390/diagnostics14161838

**Published:** 2024-08-22

**Authors:** Larisa A. Dobrynina, Elena I. Kremneva, Kamila V. Shamtieva, Anastasia A. Geints, Alexey S. Filatov, Zukhra Sh. Gadzhieva, Elena V. Gnedovskaya, Marina V. Krotenkova, Ivan I. Maximov

**Affiliations:** 1 Research Center of Neurology, 125367 Moscow, Russia; dobrla@mail.ru (L.A.D.); anastasiyatarasova75@gmail.com (A.A.G.); fil4tovmd@gmail.com (A.S.F.); gnedovskaya@mail.ru (E.V.G.); krotenkova_mrt@mail.ru (M.V.K.); 2 Department of Health and Functioning, Western Norway University of Applied Sciences (HVL), 5063 Bergen, Norway; ivan.maximov@hvl.no

**Keywords:** small vessel disease, cognitive impairment, corpus callosum, diffusion models, tract profiles

## Abstract

The cerebral small vessel disease (cSVD) is one of the main causes of vascular and mixed cognitive impairment (CI), and it is associated, in particular, with brain ageing. An understanding of structural tissue changes in an intact cerebral white matter in cSVD might allow one to develop the sensitive biomarkers for early diagnosis and monitoring of disease progression. Purpose of the study: to evaluate microstructural changes in the corpus callosum (CC) using diffusion MRI (D-MRI) approaches in cSVD patients with different severity of CI and reveal the most sensitive correlations of diffusion metrics with CI. Methods: the study included 166 cSVD patients (51.8% women; 60.4 ± 7.6 years) and 44 healthy volunteers (65.9% women; 59.6 ± 6.8 years). All subjects underwent D-MRI (3T) with signal (diffusion tensor and kurtosis) and biophysical (neurite orientation dispersion and density imaging, NODDI, white matter tract integrity, WMTI, multicompartment spherical mean technique, MC-SMT) modeling in three CC segments as well as a neuropsychological assessment. Results: in cSVD patients, microstructural changes were found in all CC segments already at the subjective CI stage, which was found to worsen into mild CI and dementia. More pronounced changes were observed in the forceps minor. Among the signal models FA, MD, MK, RD, and RK, as well as among the biophysical models, MC-SMT (EMD, ETR) and WMTI (AWF) metrics exhibited the largest area under the curve (>0.85), characterizing the loss of microstructural integrity, the severity of potential demyelination, and the proportion of intra-axonal water, respectively. **Conclusion:** the study reveals the relevance of advanced D-MRI approaches for the assessment of brain tissue changes in cSVD. The identified diffusion biomarkers could be used for the clarification and observation of CI progression.

## 1. Introduction

Age-related cerebral small vessel disease (cSVD), associated with the relevant vascular risk factors, is one of the main causes of vascular cognitive impairment (CI) and Alzheimer’s disease-related CI [1,2]. cSVD is a neurological disease with a complex pathophysiology, for example, hypoxic–ischemic white matter injuries due to arteriolosclerosis, high permeability of the blood–brain barrier (BBB) with the progressing vasogenic edema, and associated neuroinflammation [3,4,5]. Differences in the mechanisms of brain injury explain the heterogeneity of cSVD forms and the variable clinical course, including the features and rate of CI progression.

To date, there is no treatment for the cSVD disease. In turn, prevention of CI development and progression in cSVD patients is based on the correction of arterial hypertension (AH) and other vascular risk factors [6,7,8]. However, the control of risk factors at the stage of clinically significant CI (mild CI, MCI, and dementia) is less effective [6,9,10]. There are significant limitations in the dynamic assessments of the CI states in cSVD patients. These limitations are based on the slow rate of CI progression. Worth noting is that a cognitive assessment and an increasing disease severity in MRI exhibit a low sensitivity in the case of follow-up studies [10,11,12]. The difficulties associated with assessing and predicting CI progression in cSVD patients justify the search for neuroimaging markers, especially equivalents of CI in studies and then in clinical practice. 

Diffusion MRI (D-MRI) holds promises as a research tool to investigate the brain microstructure. There are a few approaches allowing the visualization of the brain tissue. The most popular approach is a diffusion tensor imaging (DTI) offering four scalar metrics. Three diffusion metrics (D-metrics) describe the diffusive properties of the tissue, namely mean, axial, and radial diffusivities (MD, AD, and RD, respectively). One more D-metric assesses the anisotropy of the underlying media and is called fractional anisotropy (FA). Previously, it has been found that cSVD severity is associated with a decrease in FA and an increase in other D-metrics in white matter hyperintensity (WMH) and the areas surrounding them, including normal-appearing white matter (NAWM) [13,14,15,16,17,18,19].

A comparison of D-metrics across different brain regions and white matter tracts has established that tissue changes in the corpus callosum (CC), in particular a decrease in FA, especially in its forceps minor and body [20,21], have the highest significance for cSVD-related CI [14,22,23].

Nevertheless, DTI is not able to explain the underlying microstructural changes. This has led to the development of advanced diffusion models. The biophysical models of D-MRI developed in recent years aim to achieve a higher biological specificity. At the moment, white matter has a commonly accepted representation based on two water compartments such as intra- and extra-axonal water [24,25,26]. Thus, the biophysical models allow to identify different types of white matter changes originating from the disease [24,25,26]. In turn, this might allow us to detect the tissue changes engendered by the complexity and heterogeneity of cSVD mechanisms. 

A limited number of studies has been published up to now that evaluate cSVD-related microstructural changes in the white matter from a biophysical point of view. A concept of “free water” in the standard diffusion model offers an explanation of persistent edema or vacuolization of myelin [27]. In another work, the neurite orientation dispersion and density imaging (NODDI) model has been used in order to evaluate the white matter changes in perivascular spaces [28]. In healthy volunteers, the free water fraction (ISO) has been shown to be smaller than that in cSVD patients. Thus, the ISO measure is consistent with the stagnation of interstitial fluid due to perivascular space dysfunction [28].

Previously, we have used signal (diffusion tensor and diffusion kurtosis) and biophysical models (NODDI, white matter tract integrity, WMTI, multicompartment spherical mean technique, MC-SMT) to assess CC microstructural integrity by tract-tracing in cSVD patients in contrast to healthy controls [29]. The most pronounced changes in microstructural integrity have been found in the CC forceps minor. Among the biophysical models, the best characteristics for cSVD (a greater area under the curve in receiver operating characteristic, ROC, analysis) have been shown by the WMTI and MC-SMT metrics, indicating an increase in extra-axonal water which might reflect the development of demyelination and tissue degeneration [29].

CC possesses the strongest association with clinical manifestations of cSVD [14,22,23]. This tract is the leading node of the neural network, providing general and high-level cognitive, behavioral, motor, and sensory integration in the brain [16,30]. The morphology of CC in cSVD has been studied previously and entails a diffuse loss of nerve fibers, demyelination, and gliosis [20,31], all of which could be connected with transependymal cerebrospinal fluid (CSF) flows. The last one plays a crucial role in the development of cSVD-associated CI [32,33,34].

## 2. Materials and Methods

The study included 166 patients (86, 51.8%, women and 80, 48.2%, men; mean age 60.4 ± 7.6 years) with diagnostic MRI signs of cSVD [35] and CI of varying severity. The control group consisted of 44 age- and sex-matched healthy volunteers (29, 65.9%, women and 15, 34.1%, men; mean age 59.6 ± 6.8 years) without neurological complaints or any MRI signs of brain pathology.

The patients did not have a hemodynamically significant atherosclerotic stenosis (>50%), a decompensated concomitant somatic pathology, or recent acute cerebrovascular accidents.

All subjects signed voluntary consent for participation in the study. The Local Ethics Committee of the Research Centre of Neurology (Moscow, Russia) approved this study. The ethics statement number is 1-8/16, dated 27 January 2016. 

The neuropsychological examination included the assessment of CI severity by the general cognitive level (Montreal cognitive function assessment scale, MoCA) [36] and independence in daily life (DSM-5) [37]. The patients were divided into three groups: group 1—dementia (MoCA < 26, loss of independence in daily life); group 2—MCI (MoCA < 26, maintaining independence in daily life); group 3—subjective CI (subCI) (MoCA ≥ 26, cognitive complaints) [36,37]. 

All patients underwent general, neurological, and neuropsychological examinations and brain MRI using a Siemens Magnetom Verio 3.0T MRI scanner (Siemens AG, Erlargen, Germany). The general brain MRI protocol included structural modes T2-weighted images, 3D T2-weighted FLAIR, 3D T1-weighted MPR, susceptibility weighted imaging, and diffusion measurements based on an echo-planar pulse sequence with three diffusion-weighted values (b = 0 s/mm^2^, 1000 s/mm^2^, 2500 s/mm^2^) for 64 directions of encoding diffusion gradients per diffusion weighting. In addition, the images with b = 0 and opposite phase encoding direction were acquired as well. Other imaging parameters were set: TR/TE = 12,600/115 ms, 100 × 100 pixels matrix, 2 × 2 × 2 mm^3^ spatial resolution.

Diffusion data preprocessing was performed using an optimized pipeline [38]. It consisted of the following steps: noise correction [39], correction of motion artifacts, external magnetic field inhomogeneities and geometric distortions caused by eddy currents (using FSL-based “topup” and “eddy” utilities) [40], correction of distortions based on the measurement of incomplete k-space (WMH-ringing artifacts) [41], and smoothing of the obtained images using the Gaussian filter with 1 mm^3^ kernel.

The diffusion maps were obtained using the MatlabR2017a software (Mathworks, MA, USA) and in-home scripts. The following metrics were derived:

- DTI: FA, MD, AD, RD;
- DKI: mean kurtosis (MK), axial kurtosis (AK), radial kurtosis (RK);
- NODDI: neurite density index (NDI), orientation dispersion index (ODI), free water fraction (ISO);
- WMTI (white matter tract integrity): axonal water fraction (AWF), axial extra-axonal diffusivity (axEAD), radial extra-axonal diffusivity (radEAD);
- MC-SMT (multicompartment spherical mean technique): intra-axonal volume fraction (INTRA), extra-axonal microscopic mean diffusivity (Extramd, EMD), extra-axonal microscopic transverse diffusivity (Extratrans, ETR).

The diffusion data were further processed using the DIPY software 1.8.0 (https://dipy.org, accessed on 21 July 2023). The processing included the following steps: exclusion of cranial bones and soft tissues of the head using a mask, construction of tractograms of the whole brain using the EuDX algorithm [42], linear registration of the obtained tractograms with the HCP842 atlas that was included in the normalized space defined by the Montreal Neurological Institute using the streamline-based linear registration (SLR) method, segmentation of the tracts of interest (forceps major, forceps minor, and body of the CC), and construction of their profiles.

Tract profiles were constructed as follows: each fiber of the studied tract was divided into 100 conditional points, where 0 corresponded to the beginning of the tract and 100 to its end. At each point, values of the selected D-metrics were calculated, and, as a result, a graph reflecting the values of this metric throughout the tract was obtained. Tract profiles for each studied metric were averaged in order to obtain the total profiles for cSVD patients and for the control group. The tract profiles were further analyzed in order to localize and determine the most affected parts of tracts.

**Figure 1:**
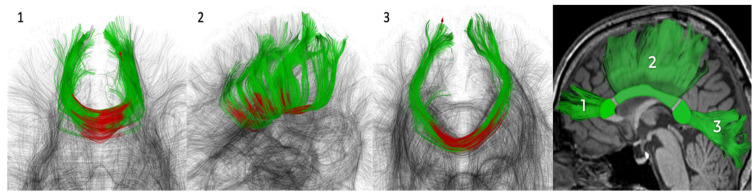
Schematic representation of the CC segmented parts: 1—forceps minor (genu), 2—body, 3—forceps major (splenium). The central segments are colored in red, the whole segmented tract is colored in green.

Inspection of the profiles of the forceps minor, forceps major, and body of the CC did not reveal areas with significant changes in all D-metrics simultaneously. Each D-metric was analyzed in its parasagittal sections (40 to 60 points, the central segment) for each region of interest (Figure 1).

The CC central segment corresponds to the longitudinally located fibers, typically without lacunas and WMH. This approach allows to avoid measurement inaccuracies caused by the intersections of conductive pathways in the semioval center and areas of complete tissue destruction.

Statistical analysis was performed using the SPSS Statistics 26.0 software (IBM, New York, NY, USA). The main descriptive statistics for categorical and ordinal variables were frequency and percentage, while, for quantitative variables, mean and standard deviation. In all cases, two-sided versions of statistical criteria were used. The null hypothesis was rejected at *p* < 0.05.

Differences between groups were determined using χ^2^, univariate analysis of variance, or the Kruskal–Wallis test, where appropriate. The ROC analysis was used in order to assess the predictive ability of individual parameters.

## 3. Results

Among the 166 cSVD patients, 40 (24.1%) (mean age 62.2 ± 7.8 years, 11, 27.5%, women), 71 (42.8%) (mean age 60.2 ± 7.5 years, 41, 57.7%, women), and 55 (33.1%) (mean age 59.4 ± 7.8 years, 32, 58.2%, women) patients had dementia, MCI, and subCI, respectively (see Table 1). Most patients had Fazekas grade 2–3 WMH: 1—18 (10.8%), 2—41 (24.7%), 3—107 (64.5%).

Almost all D-metrics in the three CC regions exhibited significant differences between the patient subgroups (subCI, MCI, dementia) and the healthy control (see Table 2 and Table 3).

The progression in CI severity from subCI to MCI and dementia was associated with a decrease in FA, MK, and RK and an increase in MD, RD, and AD, respectively.

FA, MD, RD, MK, and RK values exhibited differences between the control and the CI groups of any severity, as well as between the dementia and the MCI or subCI subgroups. AD in the forceps major and minor exhibited significant differences between the dementia and the control or subCI subjects. AK did not reveal any difference.

With CI progression, the following changes were found in NODDI: a decrease in NDI and an increase in ODI and ISO. In MC-SMT, the following changes were found: a decrease in INTRA and an increase in EMD and ETR. In WMTI, a decrease in AWF was found.

The results of the ROC analysis are summarized in Table 4. Measures of clinically significant CI with AUC > 0.8 included: FA, MD, RD, RK, INTRA, EMD, ETR, and AWF for the forceps minor, FA, MK, RK, ETR, AWF, and FA for the body, and RD, EMD, and ETR for the forceps major.

**Figure 2:**
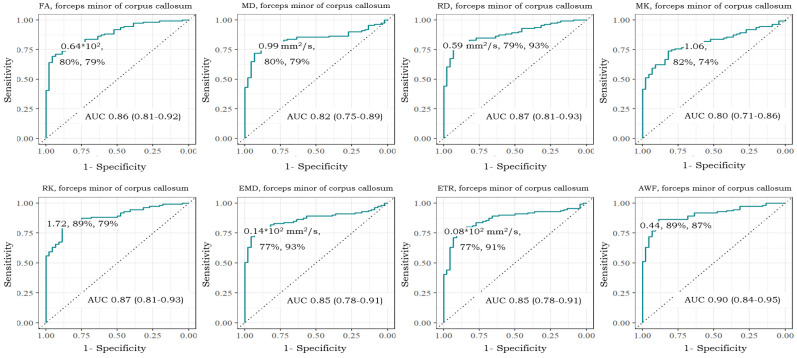
ROC curves of the highest D-metrics values (AUC > 0.8) in the forceps minor for clinically significant CI (MCI and dementia) vs. control. The green line indicates AUC.

The ROC curves of the highest D-metrics values (AUC > 0.8) in the forceps minor for clinically significant CI (MCI and dementia) are shown in Figure 2.

**Figure 3:**
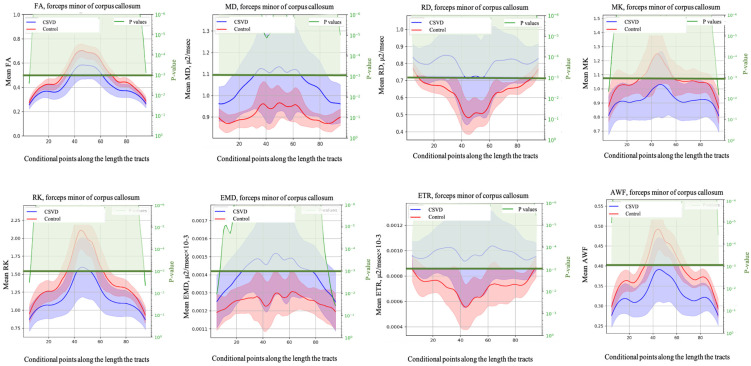
Profiles of D-metrics with AUC > 0.8 along the tract of the forceps minor for clinically significant CI vs. controls. The blue line indicates the D-metrics value for cSVD patients, the red one denotes that for the control group. The green curved line overlays the *p*-value graph. The horizontal green line indicates the significance level of *p* < 0.01. The segments of the tracts for which statistically significant differences in D-metrics values were found among the studied groups are shown in light green. The analysis was carried out from 5 to 95 conventional points of the CC tract length.

D-metrics comparisons in the forceps minor between cSVD patients and healthy controls are shown in Figure 3.

**Figure 4:**
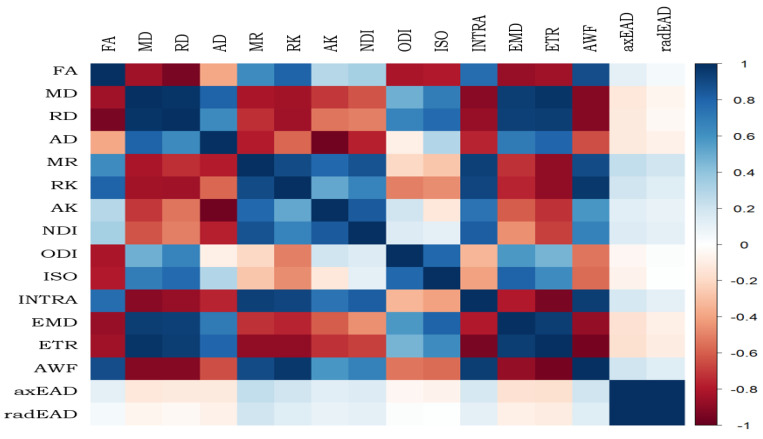
Correlations among D-metrics in the forceps minor. FA, fractional anisotropy; MD, mean diffusivity; AD, axial diffusivity; RD, radial diffusivity; MK, mean kurtosis; RK, radial kurtosis; AK, axial kurtosis; NDI, neurite density index; ODI, orientation dispersion index; ISO, free water fraction; INTRA, intra-axonal volume fraction; EMD, extra-axonal microscopic mean diffusivity; ETR, extra-axonal microscopic transverse diffusivity; AWF, axonal water fraction; axEAD, axial extra-axonal diffusivity; radEAD, radial extra-axonal diffusivity.

In order to clarify the biological significance of the used D-metrics, we performed a correlation analysis of them for the forceps minor (see Figure 4).

## 4. Discussion

Our research is proposed to determine the potential D-MRI measures of CI severity in cSVD patients. We assessed microstructural tissue changes using a series of diffusion approaches in three CC segments.

The study demonstrates that, in cSVD patients with CI, the white matter tissue changes in all the three CC segments can be detected already at the subCI stage and it further worsens into MCI and dementia. An increase in similar D-metrics in cSVD patients with CI of varying severity might indicate the presence of a common mechanism of the brain injury at all stages of the disease. These tissue changes were more pronounced in the forceps minor in contrast to the body and forceps major. This gradient of CC injury from the frontal to the parietal and occipital lobes is consistent with the cognitive profile of cSVD patients. Numerous studies have shown that, in cSVD, early and subsequently predominant disorders in the executive functions are closely related to the disintegration of the frontal lobe connectivity [43,44].

The search for the most sensitive and specific D-metric for clinically significant CI such as MCI and dementia was evaluated by ROC analysis. FA, MD, and MK exhibited the best ROC characteristics (AUC > 0.8), characterizing the overall loss of white matter integrity. Notably, the transverse diffusion indices, taking into account the Gaussian (RD) and non-Gaussian (RK) water diffusion, might represent surrogate biomarkers of demyelination. Our results are consistent with previously published studies investigating the cSVD disease. In turn, we reproduced the previous findings such as predominant injury of the CC forceps minor and body, including a decrease in FA and an increase in MD and RD [22,23,45]. In the present research, the threshold D-metrics values were determined in relation to clinically significant CI.

Among the biophysical models, the MC-SMT (EMD, ETR) and WMTI (AWF) metrics had the largest AUC (> 0.8). The correctness of MC-SMT for quantifying intra- and extra-axonal compartments was confirmed in a mouse model of tuberous sclerosis [46], while the feasibility of WMTI was confirmed in an experimental model of demyelination induced by rodent intoxication with cuprizone [47]. Following histological validation of these D-metrics, the latter can be used to interpret conditions related to myelin and axonal integrity [47,48]. It is likely that our study is the first one to use MC-SMT and WMTI in relation to cSVD. However, one should interpret our results with caution in terms of the applicability of the used D-metrics, due to their assumptions being applied in order to avoid the typical problems of standard diffusion modelling [25].

With the progression of CI, EMD and ETR increased, while AWF decreased. EMD and ETR are considered to be surrogate biomarkers of the severity of white matter injury associated with the loss of myelin [46]. It can be argued that an increase in these D-metrics, along with CI severity, corresponds to progressive myelin injury with an increase in extracellular water, a phenomenon which is consistent with morphological and experimental data on demyelination of white matter in cSVD patients [49], as well as on the predominance of demyelination compared with axonal degeneration in the CC of patients affected by Binswanger’s disease [50].

Taking into account pathomorphological data, demyelination may be a consequence of both ischemic injury due to the arteriolosclerosis and hypoxia due to edema [5,23,51]. The obtained data might indicate a high or predominant value of the latter in the white matter injury in cSVD-associated CI. The vasogenic interstitial edema in cSVD is closely associated with such recognized pathophysiological mechanisms of the disease as increased BBB permeability [3,4,5], glymphatic dysfunction with impaired brain drainage [52,53], and transependymal CSF flow [32,33,34]. Our findings relative to the extra-axonal space compartment in cSVD patients with CI should correspond to the similar results proposed by Duering and colleagues (2018) based on evaluating of the free water (FW) imaging [27]. The authors concluded that an extracellular fluid volume increase is a critical factor for the description of the brain injury in cSVD and of its clinical symptoms, rather than a change in the white matter integrity [27]. However, previously, it has been shown that white matter connectivity is consistent with the results from pathological studies [50]. The revealed decrease in AWF is significant for the evaluation of CI progression. Thus, AWF and, partially, INTRA could be considered to be surrogate markers of axon loss [54], a phenomenon which is also consistent with morphological data on diffuse loss of nerve fibers and gliosis in the CC of cSVD patients [20,31,50]. In addition, the known increase in the extra-axonal water fraction with CI progression, together with the axon loss, can also be explained by a relative decrease in the fraction of the intra-axonal water relative to the extra-axonal one.

Notably, NODDI was less sensitive with respect to the description of CI progression in contrast to previous results [28]. However, the nature of the changes obtained through NODDI metrics might correspond to those obtained through MC-SMT (EMD, ETR) and WMTI (AWF) metrics. Previously, usage of NODDI in cSVD has revealed the changes in the CC forceps minor, in particular an increase in ISO and a decrease in NDI, indicating an increase in the free water fraction and a decrease in the proportion of intra-axonal water, respectively [55]. Taking into account the higher-than-normal ISO values near the perivascular spaces in cSVD patients, Y. Jiaerken et al. (2021) suggested that these changes correspond to interstitial fluid retention [28].

Most of MC-SMT and WMTI metrics showed better performance compared to other D-metrics in the forceps minor and major, in particular with respect to cSVD patients with clinically significant CI. The revealed sensitive metrics of WMTI and MC-SMT biophysical models exhibited stronger correlations with one another and with DTI/DKI metrics. EMD and ETR exhibited strong positive correlations with MD and RD, as well as with AWF. Our data confirmed that EMD, ETR, MD, and RD can be used as surrogate markers of demyelination and changes in extracellular (interstitial) space [46,56]. Complementary D-metrics such as AWF can be used as markers of axonal degeneration [54].

The low signal-to-noise ratio of the diffusion models is, probably, the main limitation. Moreover, we revealed the high correlations of the D-metrics characterizing the axonal states with FA, a phenomenon which does not exclude degeneration, although there were no correlations with AD, a surrogate signal metric of degeneration. The impossibility of an unambiguous interpretation of these data is also indicated by the fact that D-metrics changes associated with the axon state occurred already at the subCI stage, when, as shown by histological studies, degeneration processes are not detected, but only the accumulation of extracellular water and demyelination occurs [57].

## 5. Conclusions

Diffusion MRI allowed us to reveal the tissue changes in the extra-axonal space of the CC. A combination of the conventional D-metrics from DTI and DKI and advanced biophysical models (MC SMT, WMTI, and NODDI) demonstrated a great ability to predict CI severity. As a result, a range of D-metrics based on biophysical models could be used as useful biomarkers of CI in cSVD patients to predict cSVD progression and response to treatment.

## Figures and Tables

**Table 1:** Characteristics of cSVD patients by CI severity and control.

Characteristics	Control (0) (n = 44)	SubCI (1) (n = 55)	MildCI (2) (n = 71)	Dementia (3) (n = 40)	*p*, Post-Hoc
Gender, women (n, %)	29 (65.9%)	32 (5.2%)	41 (57.7%)	11 (27.5%)	0.001
Age, years (mean ± SD)	59.6 ± 6.8	59.4 ± 7.8	60.2 ± 7.5	62.2 ± 7.8	0.007 *p*_0-3_ = 0.004
AH (n, %)	19 (43.2%)	50 (90.9%)	68 (95.8%)	40 (100%)	<0.001
Type 2 diabetes (n, %)	2 (4.5%)	9 (16.4%)	15 (21.1%)	11 (27.5%)	0.022
Smoking (n, %)	14 (31.8%)	21 (38.2%)	17 (23.9%)	12 (12%)	0.202
Hypercholesterolemia * (n, %)	7 (15.9%)	24 (43.6%)	42 (59.2%)	26 (65%)	<0.001
Obesity ** (n, %)	9 (20.5%)	16 (29.1%)	25 (35.2%)	15 (37.5%)	0.014
WMH, Fazekas scale (n, %)					<0.001
Grade 1		11 (20%)	7 (9.9%)	0	
Grade 2		17 (30.9%)	22 (30.9%)	1 (2.5%)	
Grade 3		27 (49.9%)	42 (59.2%)	39 (97.5%)	
MoCA	29 [27; 29]	27 [26; 28]	23 [21; 24]	17 [14; 20]	*p* < 0.001
					*p*_3-2_ = 0.002
					*p*_3-1,0; 2-1,0_ < 0.001
					*p*_1-0_ > 0.05

* (total cholesterol > 6.2 mmol/L or taking statins). ** body mass index > 30 kg/m^2^. Note: AH, arterial hypertension; MoCA, Montreal cognitive function assessment scale; WMH, white matter hyperintensity.

**Table 2:** D-metrics of signal models in the CC of cSVD patients with CI of varying severity vs. control.

D-metric	Control (0)	subCI (1)	MCI (2)	Dementia (3)	*p* Value	*p*, Post-Hoc
	Me [Q25%;Q75%]	Me [Q25%;Q75%]	Me [Q25%;Q75%]	Me [Q25%;Q75%]		
Diffusion tensor imaging (DTI)						
FA, fractional anisotropy						
Forceps minor	0.68 [0.65; 0.70]	0.61 [0.56; 0.64]	0.59 [0.58; 0.63]	0.51 [0.46; 0.57]	<0.001	*p*_0-1,2,3; 3-1,2_ < 0.001
Body	0.68 [0.65; 0.69]	0.61 [0.58; 0.65]	0.61 [0.59; 0.63]	0.51 [0.47; 0.53]	<0.001	*p*_0-1,2,3; 3-1,2_ ≤ 0.001
Forceps major	0.74 [0.71; 0.75]	0.68 [0.65; 0.71]	0.66 [0.65; 0.69]	0.56 [0.52; 0.58]	<0.001	*p*_0-1,2,3; 3-1,2_ = 0.001–0.003
MD, mean diffusivity						
Forceps minor	0.95 [0.93; 0.96]	1.05 [1.00; 1.10]	1.07 [1.03; 1.10]	1.21 [1.13; 1.33]	<0.001	*p*_0-1,2,3; 3-1,2_ = 0.001–0.008
Body	1.10 [1.09; 1.15]	1.19 [1.15; 1.23]	1.20 [1.16; 1.23]	1.28 [1.24; 1.33]	<0.001	*p*_0-1,2,3; 3-2_ = 0.001–0.04
Forceps major	0.95 [0.93; 0.97]	1.02 [0.99; 1.06]	1.05 [1.00; 1.12]	1.22 [1.16; 1.31]	<0.001	*p*_0-1,2,3; 3-1,2_ = 0.001–0.03
RD, radial diffusivity						
Forceps minor	0.52 [0.49; 0.54]	0.62 [0.58; 0.75]	0.64 [0.61; 0.69]	0.84 [0.72; 0.99]	<0.001	*p*_0-1,2,3; 3-1,2_ = 0.001–0.002
Body	0.61 [0.57; 0.66]	0.73 [0.67; 0.79]	0.75 [0.69; 0.80]	0.90 [0.82; 0.99]	<0.001	*p*_0-1,2,3; 3-1,2_ = 0.001–0.002
Forceps major	0.45 [0.43; 0.51]	0.54 [0.51; 0.63]	0.59 [0.54; 0.64]	0.79 [0.73; 0.85]	<0.001	*p*_0-1,2,3; 3-1,2_ = 0.001
AD, axial diffusivity						
Forceps minor	1.85 [1.76; 1.90]	1.87 [1.82; 1.94]	1.88 [1.79; 1.95]	1.96 [1.85; 2.08]	0.004	*p*_3-0_ = 0.002 *p*_3-1_ = 0.03
Body	2.13 [2.09; 2.14]	2.14 [2.09; 2.19]	2.11 [2.09; 2.17]	2.04 [1.97; 2.13]	0.132	-
Forceps major	1.91 [1.87; 1.93]	1.95 [1.89; 1.99]	1.97 [1.86; 2.06]	2.07 [1.97; 2.15]	0.002	*p*_3-0_ = 0.001 *p*_3-1_ = 0.03
Diffusion Kurtosis Imaging (DKI)						
MK, mean kurtosis						
Forceps minor	1.16 [1.11; 1.19]	1.00 [0.96; 1.07]	1.00 [0.97; 1.05]	0.89 [0.85; 0.93]	<0.001	*p*_0-1,2,3; 3-1,2_ = 0.001–0.002
Body	1.07 [1.05; 1.09]	1.00 [0.95; 1.03]	0.99 [0.95; 1.02]	0.87 [0.82; 0.90]	<0.001	*p*_0-1,2,3; 3-1,2_ ≤ 0.001
Forceps major	1.23 [1.19; 1.35]	1.12 [1.09; 1.22]	1.11 [1.05; 1.26]	0.95 [0.91; 1.02]	<0.001	*p*_0-1,2,3_ < 0.001
RK, radial kurtosis						
Forceps minor	1.91 [1.88; 1.99]	1.59 [1.48; 1.71]	1.63 [1.53; 1.69]	1.32 [1.16; 1.47]	<0.001	*p*_0-1,2,3; 3-1,2_ < 0.001
Body	1.92 [1.82; 1.99]	1.66 [1.63; 1.78]	1.69 [1.59; 1.76]	1.35 [1.30; 1.42]	<0.001	*p*_0-1,2,3; 3-1,2_ ≤ 0.001
Forceps major	2.09 [1.98; 2.17]	1.81 [1.71; 1.96]	1.82 [1.69; 1.94]	1.48 [1.37; 1.57]	<0.001	*p*_0-1,2,3; 3-1,2_ < 0.001
AK, axial kurtosis						
Forceps minor	0.66 [0.64; 0.70]	0.65 [0.61; 0.68]	0.65 [0.62; 0.69]	0.61 [0.59; 0.66]	0.065	-
Body	0.58 [0.57; 0.59]	0.58 [0.57; 0.59]	0.58 [0.57; 0.60]	0.58 [0.57; 0.63]	0.256	-
Forceps major	0.62 [0.61; 0.64]	0.62 [0.58; 0.63]	0.61 [0.59; 0.65]	0.58 [0.56; 0.63]	0.178	-

Note: FA, fractional anisotropy; MD, mean diffusivity; AD, axial diffusivity; RD, radial diffusivity; MK, mean kurtosis; RK, radial kurtosis; AK, axial kurtosis.

**Table 3:** D-metrics of the biophysical models in the CC of cSVD patients with CI of varying severity vs. control.

D-metric	Control (0)	subCI (1)	MCI (2)	Dementia (3)	*p* Value	*p*, Post-Hoc
	Me [Q25%; Q75%]	Me [Q25%; Q75%]	Me [Q25%; Q75%]	Me [Q25%; Q75%]		
NODDI, neurite orientation dispersion and density imaging						
NDI, neurite density index						
Forceps minor	0.78 [0.66; 0.85]	0.67 [0.58; 0.78]	0.72 [0.59; 0.82]	0.62 [0.53; 0.75]	<0.001	*p*_3-0_ < 0.001
Body	0.73 [0.68; 0.76]	0.68 [0.65; 0.74]	0.69 [0.64; 0.76]	0.58 [0.53; 0.68]	<0.001	*p*_3-0,1,2_ < 0.001
Forceps major	0.83 [0.73; 0.88]	0.75 [0.68; 0.86]	0.78 [0.68; 0.88]	0.67 [0.57; 0.84]	<0.001	*p*_3-0_ < 0.001 *p*_3-2_ = 0.010
ODI, orientation dispersion index						
Forceps minor	0.07 [0.06; 0.09]	0.09 [0.07; 0.12]	0.09 [0.07; 0.12]	0.10 [0.08; 0.16]	<0.001	*p*_3-0_ < 0.001
Body	0.06 [0.05; 0.07]	0.07 [0.06; 0.08]	0.06 [0.06; 0.08]	0.09 [0.07; 0.11]	<0.001	*p*_3-0_ < 0.001 *p*_3-1_ = 0.004
Forceps major	0.07 [0.06; 0.09]	0.08 [0.07; 0.10]	0.08 [0.07; 0.10]	0.10 [0.08; 0.13]	<0.001	*p*_3-0_ < 0.001 *p*_3-1_ = 0.007
ISO, free water fraction						
Forceps minor	0.18 [0.13; 0.21]	0.2 [0.17; 0.24]	0.2 [0.17; 0.28]	0.26 [0.20; 0.35]	<0.001	*p*_3-0,1_ <.001 *p*_2-0_ = 0.002
Body	0.25 [0.23; 0.30]	0.29 [0.24; 0.34]	0.29 [0.24; 0.35]	0.31 [0.25; 0.36]	0.036	-
Forceps major	0.17 [0.15; 0.22]	0.21 [0.18; 0.24]	0.23 [0.19; 0.29]	0.31 [0.26; 0.38]	<0.001	*p*_3-0,1,2; 2-0_ < 0.001
MC-SMT, multicompartment spherical mean technique						
INTRA, intra-axonal volume fraction						
Forceps minor	0.75 [0.73; 0.78]	0.65 [0.57; 0.72]	0.65 [0.60; 0.72]	0.53 [0.46; 0.61]	0.001	*p*_3-0,1,2; 2-0, 1-0_ < 0.001
Body	0.69 [0.63; 0.72]	0.63 [0.54; 0.68]	0.62 [0.57; 0.66]	0.51 [0.47; 0.58]	0.001	*p*_3-0,1,2_ < 0.001 *p* _2-0,1_ = 0.001
Forceps major	0.8 [0.76; 0.83]	0.74 [0.67; 0.78]	0.71 [0.62; 0.79]	0.59 [0.48; 0.65]	0.001	*p*_3-0,1,2; 2-0_ < 0.001
EMD, extra-axonal microscopic mean diffusivity						
Forceps minor	0.0013 [0.0012; 0.0013]	0.0014 [0.0013; 0.0016]	0.0015 [0.0013; 0.0015]	0.0016 [0.0015; 0.0018]	<0.001	*p*_3-0,1,2; 2-0,1_ < 0.001
Body	0.0015 [0.0014; 0.0016]	0.0016 [0.0015; 0.0017]	0.0016 [0.0015; 0.0017]	0.0017 [0.0016; 0.0018]	<0.001	*p*_3-0_ < 0.001 p1-0, 2-0 = 0.001
Forceps major	0.0012 [0.0011; 0.0013]	0.0013 [0.0013; 0.0015]	0.0014 [0.0013; 0.0016]	0.0017 [0.0016; 0.0018]	<0.001	*p*_3-0,1,2; 2-0_ < 0.001 *p*_1-0_ = 0.001
ETR, extra-axonal microscopic transverse diffusivity						
Forceps minor	0.0006 [0.0005; 0.0007]	0.0009 [0.0007; 0.0011]	0.0009 [0.0007; 0.0010]	0.0012 [0.0009; 0.0014]	<0.001	*p*_3-0,1,2; 0-1,2_ < 0.001
Body	0.0009 [0.0008; 0.0010]	0.0011 [0.0009; 0.0013]	0.0011 [0.0009; 0.0012]	0.0013 [0.0012; 0.0014]	<0.001	*p*_3-0,2; 1-0_ < 0.001 *p*_3-1_ = 0.002 *p*_2-0_ = 0.001
Forceps major	0.0005 [0.0004; 0.0006]	0.0007 [0.0006; 0.0009]	0.0008 [0.0006; 0.0010]	0.0012 [0.0010; 0.0014]	<0.001	*p*_3-0,1,2; 2-0_ < 0.001 *p*_1-0_ = 0.002
WMTI, white matter tract integrity						
AWF, axonal water fraction						
Forceps minor	0.46 [0.44; 0.49]	0.39 [0.34; 0.45]	0.40 [0.36; 0.43]	0.34 [0.29; 0.37]	<0.001	*p*_3-0,1,2; 0-1,2_ < 0.001
Body	0.43 [0.40; 0.45]	0.38 [0.35; 0.43]	0.38 [0.35; 0.42]	0.33 [0.30; 0.36]	<0.001	*p*_3-0,1,2; 0-1,2_ < 0.001
Forceps major	0.49 [0.46; 0.53]	0.46 [0.39; 0.50]	0.44 [0.39; 0.50]	0.36 [0.30; 0.39]	<0.001	*p*_3-0,1,2_ < 0.001 *p*_1-0_ = 0.006 *p*_2-0_ = 0.001
axEAD, axial extra-axonal diffusivity						
Forceps minor	2.66 [2.55; 2.77]	2.56 [2.45; 2.66]	2.6 [2.44; 2.71]	2.6 [2.42; 2.74]	-	-
Body	2.91 [2.82; 2.98]	2.84 [2.72; 2.93]	2.84 [2.75; 2.92]	2.64 [2.48; 2.82]	<0.001	*p*_3-0,1_ < 0.001 *p*_3-2_ = 0.001
Forceps major	2.86 [2.67; 4.00]	2.88 [2.65; 3.14]	2.91 [2.75; 15.6]	2.79 [2.58; 3.0]	-	-
radEAD, radial extra-axonal diffusivity						
Forceps minor	0.85 [0.77; 0.90]	0.95 [0.84; 1.10]	1.00 [0.90; 1.10]	1.18 [1.01; 1.39]	-	-
Body	0.96 [0.90; 1.06]	1.08 [0.99; 1.21]	1.10 [0.95; 1.20]	1.26 [1.16; 1.37]	<0.001	*p*_3-0,2_ < 0.001 *p*_1-0_ = 0.001 *p*_2-0_ = 0.002
Forceps major	0.87 [0.76; 1.09]	1.01 [0.86; 1.33]	1.09 [0.91; 2.85]	1.19 [1.06; 1.43]	-	-

Note: NDI, neurite density index; ODI, orientation dispersion index; ISO, free water fraction; INTRA, intra-axonal volume fraction; EMD, extra-axonal microscopic mean diffusivity; ETR, extra-axonal microscopic transverse diffusivity; AWF, axonal water fraction; axEAD, axial extra-axonal diffusivity; radEAD, radial extra-axonal diffusivity.

**Table 4:** AUC for the ROC curves of D-metrics in the CC for clinically significant CI (MCI and dementia) in contrast to healthy controls.

D-Metric	CC Segments		
	Forceps Minor	Body	Forceps Major
DTI			
FA	0.86 (0.81–0.92), *p* < 0.001	0.82 (0.75–0.89), *p* < 0.001	0.81 (0.74–0.88), *p* < 0.001
MD	0.82 (0.75–0.89), *p* < 0.001	0.72 (0.63–0.82), *p* < 0.001	0.786 (0.71–0.86), *p* < 0.001
AD	*p* = 0.066	*p* = 0.603	0.61 (0.52–0.70), *p* = 0.039
RD	0.86 (0.81–0.93), *p* < 0.001	0.70 (0.63–0.77), *p* < 0.001	0.83 (0.76–0.90), *p* < 0.001
DKI			
MK	0.80 (0.71–0.86), *p* < 0.001	0.81 (0.73–0.89), *p* < 0.001	0.72 (0.64–0.80), *p* < 0.001
AK	*p* = 0.778	*p* = 0.066	*p* = 0.371
RK	0.87 (0.81–0.93), *p* < 0.001	0.80 (0.73–0.89), *p* < 0.001	0.79 (0.72–0.87), *p* < 0.001
NODDI			
NDI	0.67 (0.58–0.76), *p* = 0.001	0.70 (0.62–0.78), *p* < 0.001	0.62 (0.53–0.71), *p* = 0.022
ODI	0.70 (0.61–0.78), *p* < 0.001	0.70 (0.61–0.79), *p* < 0.001	0.66 (0.60–0.76), *p* = 0.002
ISO	0.68 (0.59–0.77), *p* = 0.001	0.66 (0.57–0.75), *p* = 0.002	0.80 (0.72–0.87), *p* < 0.001
MC-SMT			
INTRA	0.67 (0.58–0.76), *p* = 0.001	0.699 (0.615–0.783), *p* < 0.001	0.620 (0.529–0.712), *p* = 0.022
EMD	0.85 (0.78–0.91), *p* < 0.001	0.78 (0.70–0.86), *p* < 0.001	0.84 (0.78–0.90), *p* < 0.001
ETR	0.85 (0.78–0.91), *p* < 0.001	0.80 (0.728–0.876), *p* < 0.001	0.83 (0.77–0.90), *p* < 0.001
WMTI			
AWF	0.89 (0.84–0.94), *p* < 0.001	0.83 (0.76–0.89), *p* < 0.001	0.78 (0.70–0.85), *p* < 0.001
axEAD	0.64 (0.55–0.73), *p* = 0.008	0.68 (0.59–0.77), *p* = 0.001	*p* = 0.815
radEAD	0.79 (0.71–0.87), *p* < 0.001	0.78 (0.71–0.86), *p* < 0.001	0.71 (0.60–0.81), *p* < 0.001

Note: FA, fractional anisotropy; MD, mean diffusivity; AD, axial diffusivity; RD, radial diffusivity; MK, mean kurtosis; RK, radial kurtosis; AK, axial kurtosis; NDI, neurite density index; ODI, orientation dispersion index; ISO, free water fraction; INTRA, intra-axonal volume fraction; EMD, extra-axonal microscopic mean diffusivity; ETR, extra-axonal microscopic transverse diffusivity; AWF, axonal water fraction; axEAD, axial extra-axonal diffusivity; radEAD, radial extra-axonal diffusivity.

## Data Availability

The data presented in this study are available upon reasonable request from the corresponding author.
